# Integrative profiling of extrachromosomal circular DNA in placenta and maternal plasma provides insights into the biology of fetal growth restriction and reveals potential biomarkers

**DOI:** 10.3389/fgene.2023.1128082

**Published:** 2023-07-05

**Authors:** Minhuan Lin, Yiqing Chen, Shuting Xia, Zhiming He, Xuegao Yu, Linhuan Huang, Shaobin Lin, Binrun Liang, Ziliang Huang, Shiqiang Mei, Dong Liu, Lingling Zheng, Yanmin Luo

**Affiliations:** ^1^Department of Obstetrics and Gynecology, The First Affiliated Hospital of Sun Yat-sen University, Guangzhou, China; ^2^Clinical Laboratory, The First Affiliated Hospital of Sun Yat-sen University, Guangzhou, China; ^3^MOE Key Laboratory of Gene Function and Regulation, State Key Laboratory for Biocontrol, School of Life Sciences, Sun Yat-sen University, Guangzhou, China

**Keywords:** extrachromosomal circular DNA, fetal growth restriction, cell-free DNA, placenta, maternal plasma

## Abstract

**Introduction:** Fetal growth restriction (FGR) is a placenta-mediated pregnancy complication that predisposes fetuses to perinatal complications. Maternal plasma cell-free DNA harbors DNA originating from placental trophoblasts, which is promising for the prenatal diagnosis and prediction of pregnancy complications. Extrachromosomal circular DNA (eccDNA) is emerging as an ideal biomarker and target for several diseases.

**Methods:** We utilized eccDNA sequencing and bioinformatic pipeline to investigate the characteristics and associations of eccDNA in placenta and maternal plasma, the role of placental eccDNA in the pathogenesis of FGR, and potential plasma eccDNA biomarkers of FGR.

**Results:** Using our bioinformatics pipelines, we identified multi-chromosomal-fragment and single-fragment eccDNA in placenta, but almost exclusively single-fragment eccDNA in maternal plasma. Relative to that in plasma, eccDNA in placenta was larger and substantially more abundant in exons, untranslated regions, promoters, repetitive elements [short interspersed nuclear elements (SINEs)/Alu, SINEs/mammalian-wide interspersed repeats, long terminal repeats/endogenous retrovirus-like elements, and single recognition particle RNA], and transcription factor binding motifs. Placental multi-chromosomal-fragment eccDNA was enriched in confident enhancer regions predicted to pertain to genes in apoptosis, energy, cell growth, and autophagy pathways. Placental eccDNA–associated genes whose abundance differed between the FGR and control groups were associated with immunity-related gene ontology (GO) terms. The combined analysis of plasma and placental eccDNA–associated genes in the FGR and control groups led to the identification of potential biomarkers that were assigned to the GO terms of the epigenetic regulation of gene expression and nutrient-related processes, respectively.

**Conclusion:** Together, our results highlight links between placenta functions and multi-chromosomal-fragment and single-fragment eccDNA. The integrative analysis of placental and plasma eccDNA confirmed the potential of these molecules as disease-specific biomarkers of FGR.

## Introduction

Fetal growth restriction (FGR), defined as an estimated fetal weight (EFW) or abdominal circumference below the 10th percentile for gestational age, occurs in up to 10% of pregnancies (Buck Louis et al., 2015; American College of Obstetricians and Gynecologists, 2021). It is a common manifestation of the “great obstetrical syndromes” (also known as placenta-mediated pregnancy complications), which correlate strongly with dysfunctional placental development and increase the risk of adverse pregnancy outcomes (Brosens et al., 2011; Pijnenborg et al., 2011; Prins et al., 2022). In placentas affected by FGR, excessive oxidative stress and inflammation damage the DNA, proteins, and lipids and induce a harmful immune response (Schoots et al., 2018). In addition, placental trophoblast components are released into the maternal circulation; fetal DNA is the second most common origin of maternal plasma cell-free DNA (cfDNA) (Wong et al., 2016). Studies have revealed the impacts of genomic, epigenomic, and transcriptomic alterations in the pathogenesis of FGR, providing opportunities to improve the treatment of this condition (Salmeri et al., 2022). In addition, cfDNA has been shown to be a promising biomarker for the prediction of placenta-mediated pregnancy complications (Guo et al., 2020). However, much more investigation of this complex disease is needed.

The term “extrachromosomal circular DNA” (eccDNA) refers to a heterogeneous group of DNA circles originating from chromosomes. It was first reported in 1965 and gradually determined to be functional, but research on eccDNA commenced only recently with technological advancements, particularly the development of high-throughput sequencing and bioinformatics in the last decade (Cox et al., 1965; Lubs and Salmon, 1965). EccDNA may arise from processes such as DNA damage repair, replication, transcription, and transposition, and thus is enriched in protein-coding exons, untranslated regions (UTRs), and promoters (Schmidt et al., 2009; Kumar et al., 2017). Cancer ecDNA is larger than other forms, ranging from hundreds of kilobases to several megabases, and harbors oncogenes that drive rapid tumor evolution. Among other types of eccDNA, telomeric circles may promote alternative telomere lengthening, extrachromosomal ribosomal DNA circles may trigger cell senescence, and *HTA2-HTB2* eccDNA may compensate deleted genes (Paulsen et al., 2018). The characteristics of plasma eccDNA in the context of lung adenocarcinoma (Wu et al., 2022a) and urinary cell-free eccDNA in the context of advanced chronic kidney disease (Lv et al., 2022), and their potential roles as biomarkers, have been explored. However, many questions about the broader roles of eccDNA in diverse tissues remain unanswered.

As the placenta has a mutational genomic landscape similar to that of childhood cancer, in terms of the extensive occurrence of mutagenesis (Coorens et al., 2021), and as genomic DNA recombination contributes to eccDNA generation (Wu et al., 2022b), we conducted this study to characterize the underlying relationships between placenta-mediated pregnancy complications such as FGR and eccDNA spectrum shift. In addition, as placenta-derived eccDNA accounts for about 6%–17% of all eccDNA in maternal plasma (Sin et al., 2020), we investigated the dynamics of maternal plasma eccDNA in the context of pregnancy complications. We used eccDNA sequencing to explore the eccDNA landscape in placentas and maternal plasma from pregnancies with FGR, characterized the functional linkage of eccDNA, documented significant changes in placental eccDNA from pregnancies with FGR, and explored potential biomarkers of FGR.

## Materials and methods

### Patient recruitment and sample collection

The Ethics Committee of the First Affiliated Hospital of Sun Yat-Sen University approved this study (no. [2021]210–2) and all participants provided written informed consent. Two groups of pregnant women were recruited during their maternity ward stays for delivery at the First Affiliated Hospital of Sun Yat-Sen University. Women with singleton pregnancies without complications such as FGR, hypertensive disorders, diabetes (pre-gestational or gestational), cancer, autoimmune diseases, kidney diseases, preterm delivery, maternal or fetal chromosomal abnormalities, fetal malformation, or any other major disease served as the control group. Women with singleton pregnancies that satisfied the criteria for FGR and had no other major comorbidity were enrolled in the FGR group. As the study protocol was submitted at the end of 2020, FGR was defined, based on the 2019 American College of Obstetricians and Gynecologists practice bulletin, by an ultrasound record of EFW for gestational age below the 10th percentile on the Asian fetal growth curve (Leung et al., 2008; American College of Obstetricians and Gynecologists Committee on Practice Bulletins—Obstetrics and the Society for Maternal-Fetal Medicine, 2019). In the absence of Doppler-detected umbilical artery abnormality, induced labor was indicated at 37–37^6/7^ weeks for EFWs below the third percentile and at 38–39^6/7^ weeks for EFWs between the third and 10th percentiles (American College of Obstetricians and Gynecologists Committee on Practice Bulletins—Obstetrics and the Society for Maternal-Fetal Medicine, 2019). Fourteen pregnant women were enrolled in the study; maternal prenatal plasma and placental samples were collected from three FGR and three control cases, and only placental tissue was collected from the remaining four FGR and four control cases. Peripheral venous EDTA blood samples were collected at admission (37^5/7^–40^2/7^ gestational weeks) and centrifuged at 1,600 × g for 10 min at 4°C; the plasma was centrifuged at 16,000 × g and 4°C for an additional 10 min (Sin et al., 2020). Placental biopsy samples were collected immediately after birth. Each placenta was sampled at sites around the umbilical cord insertion after the removal of the decidua and amnion. Core chorion tissues were dissected and then snap-frozen in liquid nitrogen and later stored at −80°C, or immersed in 10% buffered formalin overnight and embedded in paraffin.

### DNA library preparation and sequencing

Three FGR and three control samples were used for sequencing. The protocol for DNA library preparation was based on the tagmentation-based method for plasma described by Sin et al. (2020) and the rolling circle amplification (RCA)-based method (commonly called “Circle-Seq”) for placenta described by Møller et al. (Møller et al., 2016; Møller et al., 2018). Sin et al. (2022) reported that similar eccDNA size distributions in sample replicates were obtained with the tagmentation- and RCA-based approaches. Briefly, circulating DNA was extracted from 5 mL of each plasma sample using a QIAamp circulating nucleic acid kit (Qiagen, Germany) according to the manufacturer’s protocol. Plasma circular DNA was enriched by exonuclease V (New England Biolabs, United States) digestion of linear DNA at 37°C and then purified using the MinElute reaction cleanup kit (Qiagen). DNA libraries were constructed using the Nextera XT DNA library preparation kit (Illumina, United States). For placental samples, total DNA was extracted from 150 mg of each sample using a MagAttract HMW DNA kit (Qiagen). Then, the circular DNA was enriched using a Plasmid Mini AX kit (A&A Biotechnology, Poland). After digestion of the mitochondrial DNA with endonuclease MssI (GTTT ^AAAC site cutting; Thermo Fisher, United States) for 16 h, residual linear DNA was removed with exonuclease (Plasmid-Safe ATP-dependent DNase; Epicentre, United States). The removal of linear DNA was confirmed by quantitative PCR of the COX5B gene. EccDNA-enriched samples were used as templates for RCA with phi29 DNA polymerase (REPLI-g midi kit; Qiagen). The amplified circular DNA was cleaned (AMPure XP beads; Beckman Coulter, United States) and sheared by sonication (Bioruptor, Belgium) to average fragment sizes of 200–300 bp. Libraries for next-generation sequencing were prepared using the NEBNext Ultra DNA library kit for Illumina (New England Biolabs) according to the manufacturer’s protocol. All libraries were sequenced with an Illumina Novaseq 6,000 device using paired-end 150.

### Identification of eccDNA from multiple sources

**FIGURE 1:**
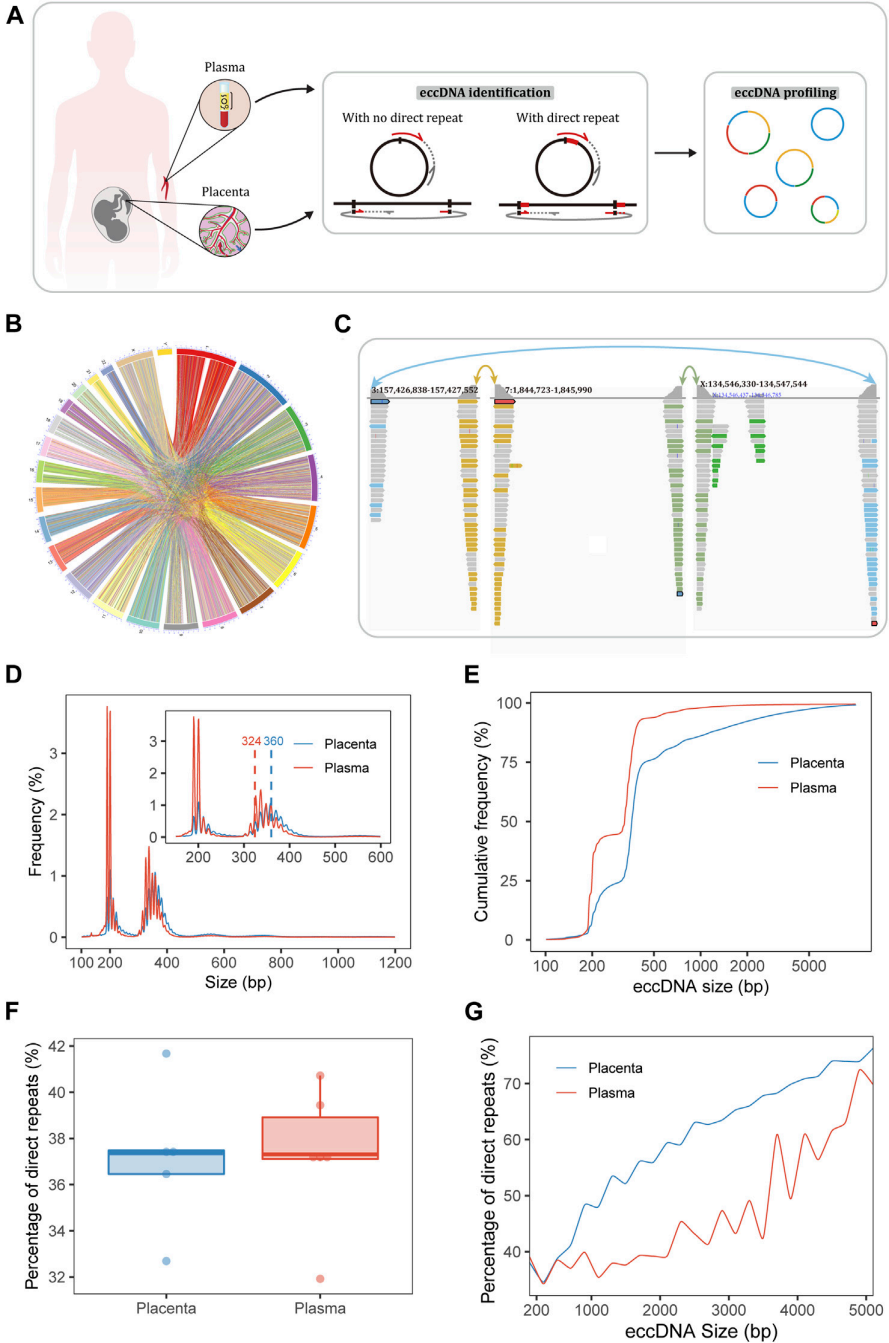
Sequencing and identification of eccDNA. **(A)** Workflow of sampling and eccDNA identification. The Circle_finder algorithm was optimized for the identification of eccDNA: we corrected eccDNA loci at base-pair resolution as when a direct repeat at the junction (red lines on the circles) was mapped to the genome, the direct repeat on a split read was mapped twice using alignment software; we also developed a script to extract confident regions with both ends supported by split reads from different chromosomes. **(B)** Circle plot of pairs of placental eccDNA genomic breakpoints joined by fragments from different chromosomes. **(C)** Example of three-fragment placental eccDNA with supportive confident split reads visualized with the IGV. **(D)** Size distributions of eccDNA identified in the placenta and maternal plasma from FGR and control cases. The blue and red dashed lines mark the median sizes of placental and plasma eccDNA. **(E)** Cumulative frequency plots of placental and maternal plasma eccDNA. **(F)** Percentages of flanking direct repeats of eccDNA from placenta and maternal plasma [defined as the short sequences at the starts of the circles and genomic copies flanking the ends of the circles **(A)**]. **(G)** Percentages of flanking direct repeats of eccDNA across sizes in placenta and maternal plasma.

After checking the read quality with FastQC v0.11.9 (Babraham Institute, United Kingdom), low-quality reads and adapters were trimmed with Trimmomatic v0.39 (Bolger et al., 2014) using the palindrome mode, with the minimum length of adapter to be removed set at one base and both reads retained. The clean reads were then aligned to all chromosomes and mitochondrial DNA using the bwa-mem2 program (Vasimuddin et al., 2019). We tested several algorithms for eccDNA identification [Circle-Map (Prada-Luengo et al., 2019), ecc_finder (Zhang et al., 2021a), AmpliconArchitect (Deshpande et al., 2019), and two scripts from Circle_finder (Kumar et al., 2017)], and preliminarily validated the results by visualization with the IGV (Robinson et al., 2011). The bwa-mem-samblaster.sh Circle_finder pipeline was deemed optimal and modified for our analysis. The core function of this algorithm is to collect all the read pairs satisfying the following criteria: 1) one read of a pair is uniquely and contiguously mapped, representing a fragment of the body of the candidate eccDNA; and 2) the other read is a split read with discordant mapping that flanks the mapped read, representing the eccDNA junction (Kumar et al., 2020). The start of split read mapping was annotated as the starting position of the candidate eccDNA, and the end of the second split read mapping was annotated as the end position. We made the following major changes to the algorithm: 1) The “--acceptDupMarks” option of Samblaster (Faust and Hall, 2014) (a program for the outputting of discordant and split read pairs) was used to accept duplicate marks to allow the full discovery of all junctions; and 2) junction position shifting due to direct repeats was adjusted at base-pair resolution by correcting the two different mapping loci of a single split read (Figure 1A). As multi-chromosomal-fragment eccDNA has been detected by nanopore sequencing (Wang et al., 2021), we also developed a script to extract confident regions with 100% genomic coverage and both ends supported by split reads from different chromosomes. Because placental eccDNA from control case 3 lacked the size features apparent in other placental and plasma samples, we assumed a significant loss of eccDNA molecules had occurred during the experiments and excluded this sample from the analysis.

### Genome-wide analysis of eccDNA loci

To enable the direct comparison of eccDNA genome distributions in placenta and plasma, we partitioned the whole genome into non-overlapping 1-Mb bins and used Bedtools (Quinlan and Hall, 2010) to count the placental and plasma eccDNA in each bin. Then, the counts were normalized according to their total numbers and plotted with RIdeogram (Hao et al., 2020). The Kolmogorov–Smirnov test was used to compare the genome distribution patterns of placental and plasma eccDNA. We further mapped eccDNA loci to genic and repetitive structures. Gene annotation, CpG island data, and repeat elements were obtained from Ensembl GRCh38.104 (Ensembl, 2021) and the University of California at Santa Cruz (UCSC) database (Navarro Gonzalez et al., 2021). Expanding regions were defined as promoter regions (2 kb upstream of genes), immediate downstream regions (2 kb downstream of genes), upstream and downstream CpG island regions (2 kb upstream and downstream, respectively, of CpG islands), distal intergenic regions (chromosomal regions reducing genic and aforementioned regions). EccDNA junction loci mapped to these genomic regions were counted. The count of annotated elements was normalized by the total junction count for each sample and the coverage of each element over the whole genome to identify eccDNA generation hot spots and correlations with biological functions. EccDNA direct repeats were defined as short sequences at the starts of the circles and genomic copies flanking the ends of the circles (Shibata et al., 2012). The “direct.repeat.finder1.c” function of the Circle_finder algorithm (Kumar et al., 2017) was used to search for eccDNA breakpoint direct repeats.

### Transcription factor motif enrichment

Known motif enrichment was performed an analysis of motif enrichment (AME) with the MEME Suite Software 5.4.1 (Bailey et al., 2015), with shuffled input sequences used as controls. Motifs used for the analysis were obtained from the latest version of JASPAR-JASPAR2022_CORE_vertebrates_non-redundant_v2. meme (Castro-Mondragon et al., 2022). EccDNA breakpoint flanking regions were defined as 50 bp of each end of the eccDNA and extending 100 bp from the start and end breakpoints of eccDNA to form a 150-bp sequence. Fisher’s exact test was used to test the significance of motif enrichment and the E value threshold for motif reporting was set to 10. Using the jaspar_enrichment tool (Castro-Mondragon et al., 2022), we also explored the differential enrichment of transcription factor binding motifs between placental and plasma eccDNA and of placental eccDNA between the FGR and control groups.

### Examination of interchromosomal joined region overlapping with enhancers

To explore the relationship between enhancers and multi-chromosomal joined fragments, we downloaded data on enhancers in placenta supported by multiple high-throughput experimental datasets from EnhancerAtlas 2.0 (EnhancerAtlas, 2020; Gao and Qian, 2020) and converted the BED files from hg19 to hg38 using the UCSC liftOver tool. We then counted unique multi-chromosomal joined regions that intersected with these enhancer regions, and normalized the count according to the total number of unique regions and enhancer coverage. To determine whether the normalized enhancer count was above average, we randomly generated a group of regions with the same length as the original regions for each sample using a modified script based on CircleAnalysis (Tatman and Black, 2022; UCDenver, 2022) and compared their normalized enhancer counts. We also used the prediction of placenta enhancer–gene interactions from EnhancerAtlas 2.0 to investigate the functions of genes associated with these enhancers (Gao and Qian, 2020).

### Identification of differentially abundant placental eccDNA–associated genes

The abundance of eccDNA-associated genes in each placenta sample was counted using supportive split reads. After normalization according to the total split reads in each sample, edgeR (Robinson et al., 2010) was used for differential abundance analysis to identify key eccDNA-associated genes involved in the pathogenesis of FGR. To facilitate the elucidation of the biological roles of genes with significant differential abundance, ClusterProfiler was applied to identify significant gene ontology (GO) categories (Ashburner et al., 2000; Wu et al., 2021). Results with false discovery rates (FDRs) < 0.05 were considered to be significant.

### Outward-directed polymerase chain reaction (PCR)

Placental eccDNA–associated genes with significant differential abundance were intersected with eccDNA loci present in at least two samples, and the top 10 eccDNA loci were examined by inverse PCR. Outward-directed primers were designed using Primer Premier 5.0 (PREMIER Biosoft, United States) to yield products across the eccDNA junctions. Each 50-ul PCR system included 60 ng φ29-amplified template, 500 nM primer, and 15 μL NEBNext high-fidelity PCR master mix (New England Biolabs), and 35 PCR cycles were performed. The PCR products were visualized by 2% agarose gel electrophoresis and purified using a QIAEX II gel extraction kit (Qiagen). The junction sites of the target products were analyzed by Sanger sequencing.

### Fluorescence *in situ* hybridization (FISH)

FISH was performed with formalin-fixed paraffin-embedded (FFPE) placenta specimens from four FGR and four control cases. Multiple Cy3-labeled probes specific to the junction sites of [*SBF1^circle 50,447,497–50,447,834^*] were designed and synthesized by RiboBio (China). FISH was performed using a RiboBio kit according to the manufacturer’s instructions with slight modification. In brief, 5-µm-thick FFPE sections were deparaffinized and rehydrated before pretreatment with protease K. The sections and probes were then co-denatured at 75°C for 5 min and hybridized overnight at 37°C. The sections were washed and counterstained with 4,6-diamino-2phenylindole. Fluorescence images of five randomly chosen areas of each slice were taken and evaluated using Fiji software (Schindelin et al., 2012); the integrated fluorescence density was measured and a mean value was calculated for each sample and compared between groups.

### Analysis of differentially abundant plasma eccDNA

We combined a plasma eccDNA tagmentation-based sequencing dataset containing data from five healthy pregnant women (accession no. EGAS00001003827; https://ega-archive.org/datasets/EGAD00001005286) (Sin et al., 2020) with our data to explore the differential abundance of plasma eccDNA between the FGR and control groups. Plasma eccDNA breakpoint flanking regions from our sequencing data (*n* = 6) and the public dataset (*n* = 5) were merged. For each sample, the numbers of split reads residing in these merged regions were determined and normalized according to the total split reads. Differential regions (*p* < 0.05) were identified with edgeR (Robinson et al., 2010) and subjected to AME (Bailey et al., 2015). Regions with known motifs were submitted to the Genomic Regions Enrichment of Annotations Tool (GREAT) (McLean et al., 2010) for potential functional interpretation; proximal and distal cis-regulatory regions are assigned to genes using a binomial test and then the enriched genes are assigned GO terms. Results with FDRs < 0.05 were considered to be significant. Next, we identified unique eccDNA molecules present in at least two samples in the FGR and control groups separately and classified them as highly abundant in the respective groups. We then used the GREAT to annotate these loci to genes, which were labeled highly abundant plasma eccDNA–associated genes for each group and intersected separately with our previous data on differentially abundant placental eccDNA–associated genes to call unique biomarkers.

### Statistical analysis

All statistical analyses were performed using R-4.1 (Lucent Technologies, United States). The two-tailed Wilcoxon rank-sum test was used for comparisons between groups, unless otherwise indicated. Statistical significance was defined as *p* < 0.05.

## Results

### Multi-chromosomal-fragment eccDNA is common in the placenta

Our experimental approaches for eccDNA enrichment and identification are illustrated in Figure 1A and a flowchart of the study design is shown in Supplementary Figure S1. Clinical details of the patients (e.g., age, gestational age at the times of sampling and delivery, birth weight, birth weight percentile) and mapping statistics are provided in Supplementary Tables S1, S2. Using our modified pipelines based on Circle_finder (Kumar et al., 2017), we identified abundant multi-chromosomal-fragment and single-fragment eccDNA molecules in the placenta; multi-chromosomal-fragment eccDNA molecules were rare in maternal plasma. Medians of 3,107,864 (range, 1,093,812–3,634,082) and 84,776 (range, 73,758–132,654) confident two-chromosomal split reads were extracted from the placenta and plasma, respectively, comprising 1.3%–17.5% and 0.7%–1.4%, respectively, of the total split reads. In the placenta and plasma, medians of 10,273 (range, 3,988–27,413) and 484 (range, 383–1,181) two-fragment eccDNA molecules and 436,259 (range, 304,689–600,692) and 211,403.5 (range, 103,152–83,000) single-fragment eccDNA molecules, respectively, were identified (Supplementary Table S2). Confident two-chromosomal split reads from case FGR1 are summarized in Figure 1B and relative frequencies of conjoining between chromosomes are shown in Supplementary Figure S2. Examples of placental three-fragment eccDNA and plasma two-fragment eccDNA, visualized with the Integrative Genomics Viewer (IGV) (Robinson et al., 2011), are shown in Figure 1C; Supplementary Figure S3, respectively.

### EccDNA is larger in placenta than in plasma

EccDNA in the placenta and plasma had bimodal size distributions at around 201 and 348 bp, respectively (Figure 1D; Supplementary Figure S4). The top three size peaks were at 201, 358, and 348 bp in placenta and 190, 201, and 337 bp in plasma. The median eccDNA sizes in placenta and plasma were 360 and 324 bp, respectively (*p* = 0.005). The plotting of cumulative frequencies demonstrated that eccDNA was larger in the placenta than in plasma and that 90% of eccDNA molecules were shorter than 1,526 bp in placenta and 387 bp in plasma (Figure 1E).

Given this significant difference in size, the breakpoint properties of eccDNA in placenta and plasma may also differ. Thus, we explored the end-nucleotide and direct-repeat features of eccDNA. The percentages of T end nucleotides in placental eccDNA of the sizes < 2,000, 2,000–3,000, and > 3,000 bp were about 18%, 25%, and 27%, respectively; those in plasma eccDNA of the sizes ≤3,000 and > 3,000 were about 18% and 24%, respectively (Supplementary Figure S5). The percentage of direct repeats (2∼ bp) ranged from 31.9% to 41.7% (Figure 1F) and increased with eccDNA length (Kolmogorov–Smirnov test, *p* < 0.001, D = 0.47; Figure 1G). The percentage of 4∼-bp direct repeats in placental eccDNA was significantly larger than that in plasma eccDNA (Supplementary Figure S6). Taken together, these results indicate that the mechanisms of placental and plasma eccDNA generation differ and that microhomology is required for longer fragments to form eccDNA.

### Placental eccDNA is more enriched in functionally important regions

**FIGURE 2:**
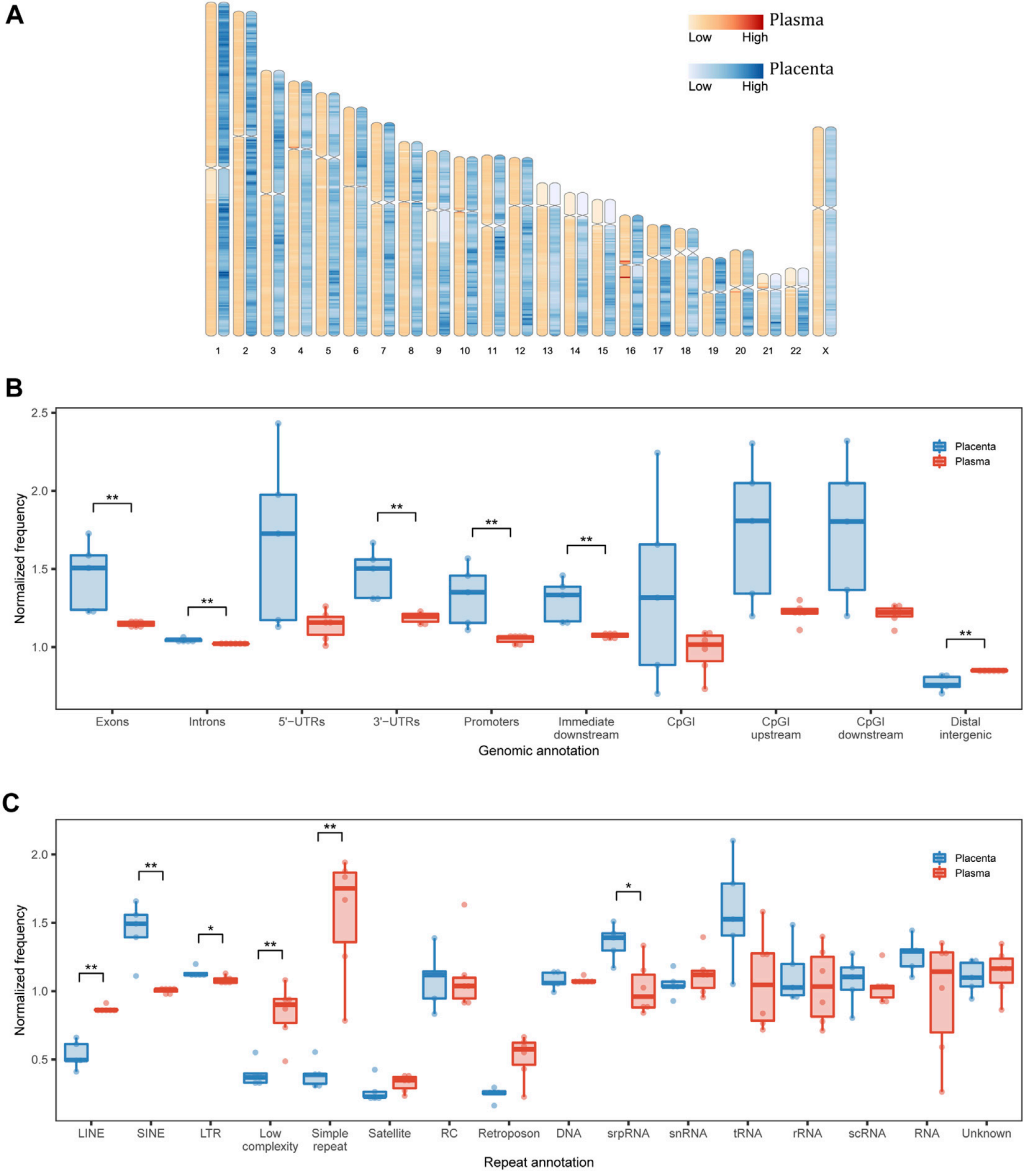
Genomic annotation of eccDNA. **(A)** Genomic distribution of eccDNA in each 1-Mb bin. **(B)** Structural and **(C)** repetitive annotations of eccDNA breakpoints in placenta and maternal plasma. UTR, untranslated region; CpGI, CpG island; LINE, long interspersed nuclear element; SINE, short interspersed nuclear element; LTR, long terminal repeat; RC, rolling circle; srpRNA, signal recognition particle RNA; scRNA, small cytoplasmic RNA; snRNA, small nuclear RNA. *p < 0.05, **p < 0.01.

To determine whether eccDNA generation is random or related to functional activities, we explored the genomic distribution and structural annotations of eccDNA. Placental eccDNA had a substantially greater abundance of some regions, whereas plasma eccDNA showed more even distribution across the whole genome (Kolmogorov–Smirnov test, *p* < 0.001, D = 0.31; Figure 2A), indicating the existence of distinct eccDNA molecular spectra correlated with the tissue of origin.

We then inspected the genomic structural annotations of single-fragment eccDNA. Placental and plasma eccDNA were enriched in 5′ UTRs, exonic regions, and CpG island regions. Compared with plasma eccDNA, placental eccDNA was significantly enriched in exonic regions, intronic regions, 3′ UTRs, promoter regions, and immediate downstream regions and had fewer distal intergenic regions (Figure 2B). The annotation of multi-chromosomal-fragment eccDNA was very similar to that of single-fragment eccDNA (Supplementary Figure S7A). The enrichment in functional regions suggests that eccDNA generation is related more closely to transcription activities in placenta than in plasma, although the normalized genomic coverage of elements did not differ significantly between the FGR and control groups (Supplementary Figures S7B, C).

As repeats favor eccDNA formation (Birkbak et al., 2012) and cause disease through a variety of mechanisms (Zhang et al., 2021b), we examined repeat annotations in eccDNA for further functional interpretation. The normalized genomic coverage of repeat elements differed significantly between placental and plasma eccDNA in terms of long interspersed nuclear elements, short interspersed nuclear elements (SINEs), long terminal repeats (LTRs), low complexity, and simple repeat and signal recognition particle RNA (srpRNA; Figure 2C; Supplementary Figure S8). Specifically, placental eccDNA was more enriched in SINE/Alu, SINE/mammalian-wide interspersed repeat (MIR), LTR/endogenous retrovirus-like (ERVL), and srpRNA elements (Supplementary Figure S9), and plasma eccDNA was more enriched in simple repeats. These findings suggest that the activities of repeat elements also shape the eccDNA atlas, depending on the tissue of origin.

### Placental eccDNA is more enriched in transcription factor binding motifs

**FIGURE 3:**
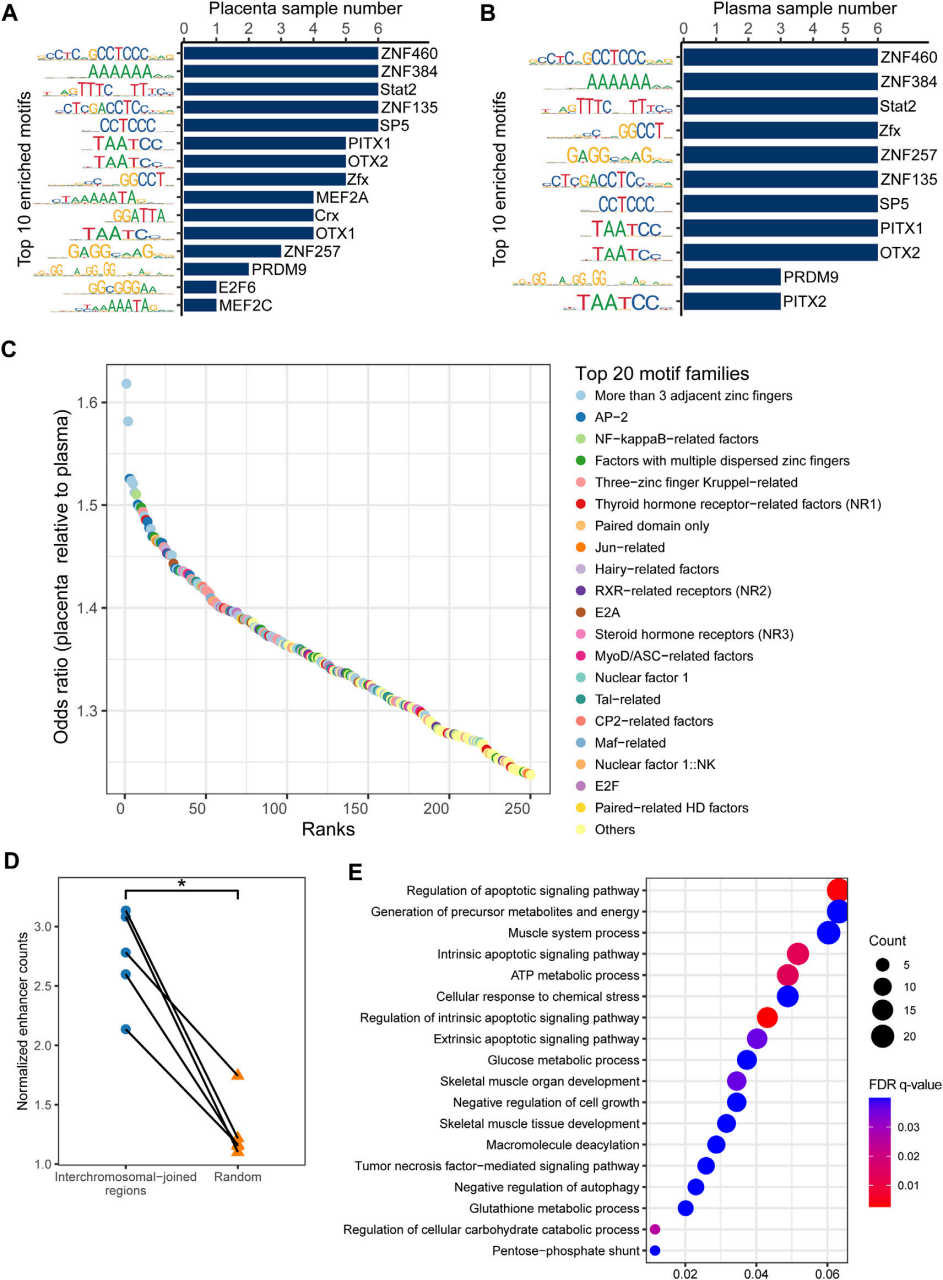
Enriched transcription factor binding motifs for eccDNA flanking regions. Top 10 motifs in **(A)** placenta and **(B)** maternal plasma. **(C)** Top 20 significantly enriched transcription factor families in placental eccDNA relative to plasma eccDNA. **(D)** Normalized enhancer counts for confident multi-chromosomal joined regions and randomly generated regions. One-tailed Wilcoxon signed-rank test, *p* = 0.031. **(E)** GO terms enriched in multi-chromosomal-fragment eccDNA–associated genes. *p < 0.05.

CfDNA fragmentation patterns have been demonstrated to reflect the accessibility of transcription factor binding sites (TFBSs) and to shift with disease occurrence (Ulz et al., 2019), and the same may be true for eccDNA. We performed an analysis of motif enrichment (AME) (Bailey et al., 2015) in eccDNA flanking regions of the placenta and plasma samples. ZNF460, ZNF384, Stat2, ZNF135, and SP5 were the most commonly enriched motifs in all samples (Figures 3A, B), reflecting common eccDNA generation hotspots. Using the jaspar_enrichment tool (Castro-Mondragon et al., 2022), we found significant enrichment of flanking motifs in placental eccDNA relative to plasma eccDNA. The top enriched transcription factor families included more than three adjacent zinc fingers, activating enhancer-binding protein 2 (AP-2), and nuclear factor-kappa B (NF-κB)– and Jun-related factors (Figure 3C). In addition, placental eccDNA from FGR cases was enriched in paired-related homeodomain (HD) factors, NK homeobox, three-amino-acid loop extension (TALE)-type HD factors, and forkhead box (FOX) compared with controls (Supplementary Figure S10). These findings suggest that transcription factor–binding activities are associated with eccDNA formation and its potential to be transcribed and to function independently of chromosomes.

### Multi-chromosomal-fragment eccDNA is associated with enhancers

As cancer ecDNA is known to function as a mobile transcriptional enhancer to amplify chromosomal transcription (Zhu et al., 2021), we hypothesized that placental multi-chromosomal-fragment eccDNA would be enriched in enhancers and that the multi-chromosomal junctions were partly the result of inter-chromosomal and eccDNA-chromosomal spatial proximity. Confident placenta enhancer regions from EnhancerAtlas 2.0 (Gao and Qian, 2020) were intersected with multi-chromosomal joined regions. The normalized enhancer count for confident multi-chromosomal joined regions was 2.1–3.1, significantly higher than that for randomly generated regions (one-tailed Wilcoxon signed-rank test, *p* = 0.03; Figure 3D). In addition, genes associated with these enhancers in more than one sample were enriched in GO terms such as the regulation of the apoptotic signaling pathway, generation of precursor metabolites and energy, negative regulation of cell growth, and negative regulation of autophagy (Figure 3E). These results suggest that placental eccDNA very likely functions as a transcriptional enhancer.

### Differentially abundant placental eccDNA–associated genes from FGR cases show enrichment in immunity-related GO terms

**FIGURE 4:**
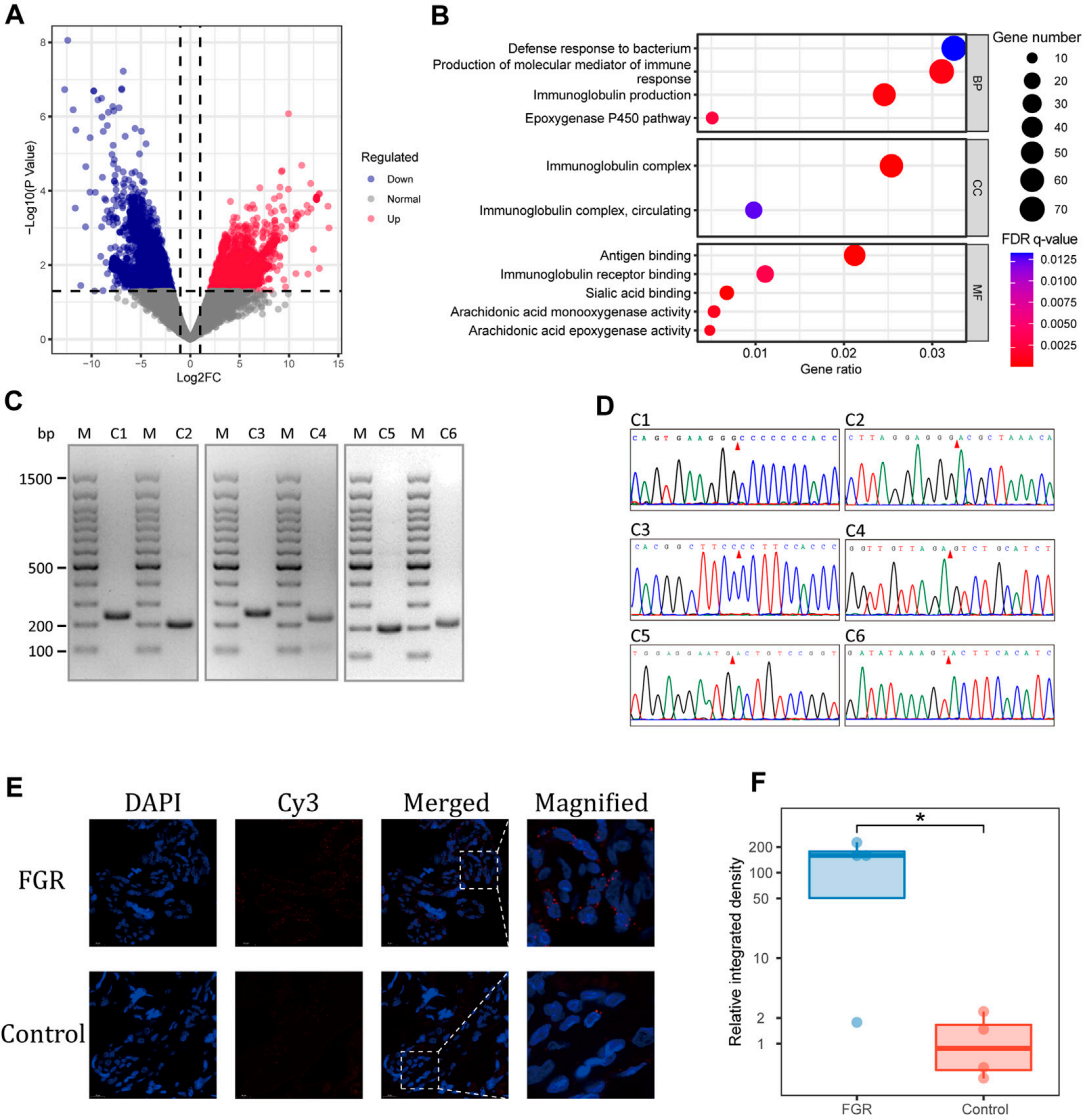
Differential abundance of placental eccDNA–associated genes. **(A)** Volcano plot of the differential abundance of placental eccDNA–associated genes between the FGR and control groups. **(B)** GO terms enriched for these genes. **(C)** PCR validation of placental eccDNA. The M lanes represent the molecular weight markers and lanes C1–C6 represent the six placental eccDNA molecules for which details are provided in Supplementary Table S3. **(D)** Junctional sites validated by Sanger sequencing of PCR products. **(E)** Representative images of FISH assays of [*SBF1^circle 50,447,497–50,447,834^*] in placenta. **(F)** Boxplot of the relative integrative density of FISH signals in placenta of four FGR cases and four control cases. *p < 0.05.

The enrichment of eccDNA in functional genic structures, repeat elements, and cis-regulatory elements demonstrated its functional potential. We performed a differential abundance analysis of placental eccDNA–associated genes from the FGR and control groups. In total, 6,129 differentially abundant eccDNA-associated genes showed enrichment in GO terms including molecular mediator of immune response and immunoglobulin production, circulating immunoglobulin complex, and the epoxygenase P450 pathway (Figures 4A, B).

To confirm the findings of the bioinformatics analysis, outward PCR was performed on 10 differentially abundant placental eccDNA molecules from the same samples used for deep sequencing. Six of the candidates were confirmed to have the expected sizes by agarose gel electrophoresis and validated by Sanger sequencing (Figures 4C, D; Supplementary Table S3). Among them, [*SBF1* ^*circle 50,447,497–50,447,834*^], [*CDK11B* ^*circle 1,637,722–1,638,075*^], [*BLK* ^*circle 11,514,374–11,514,746*^], and [ENSG00000254775^*circle 60,055,796–60,056,151*^] were highly abundant and [*BNIP3L* ^*circle 26,465,792–26,466,176*^] and [ENSG00000286489^*circle 101,699,278–101,699,642*^] had low abundance in placental samples from the FGR group. Among the differentially abundant eccDNA molecules in placenta from FGR cases, [*SBF1* ^*circle 50,447,497–50,447,834*^] was conformably present in three cases and thus was chosen for further investigation. FFPE placental samples from another eight FGR and control cases were subjected to FISH, with probes targeting junctions of [*SBF1* ^*circle 50,447,497–50,447,834*^] (Figure 4E). The semi-quantification of the integrated density of the probe signals confirmed that placentas from FGR cases had a significantly elevated abundance of [*SBF1* ^*circle 50,447,497–50,447,834*^] (one-tailed Wilcoxon rank-sum test, *p* = 0.03; Figure 4F).

### Plasma eccDNA is a potential novel biomarker of FGR

**FIGURE 5:**
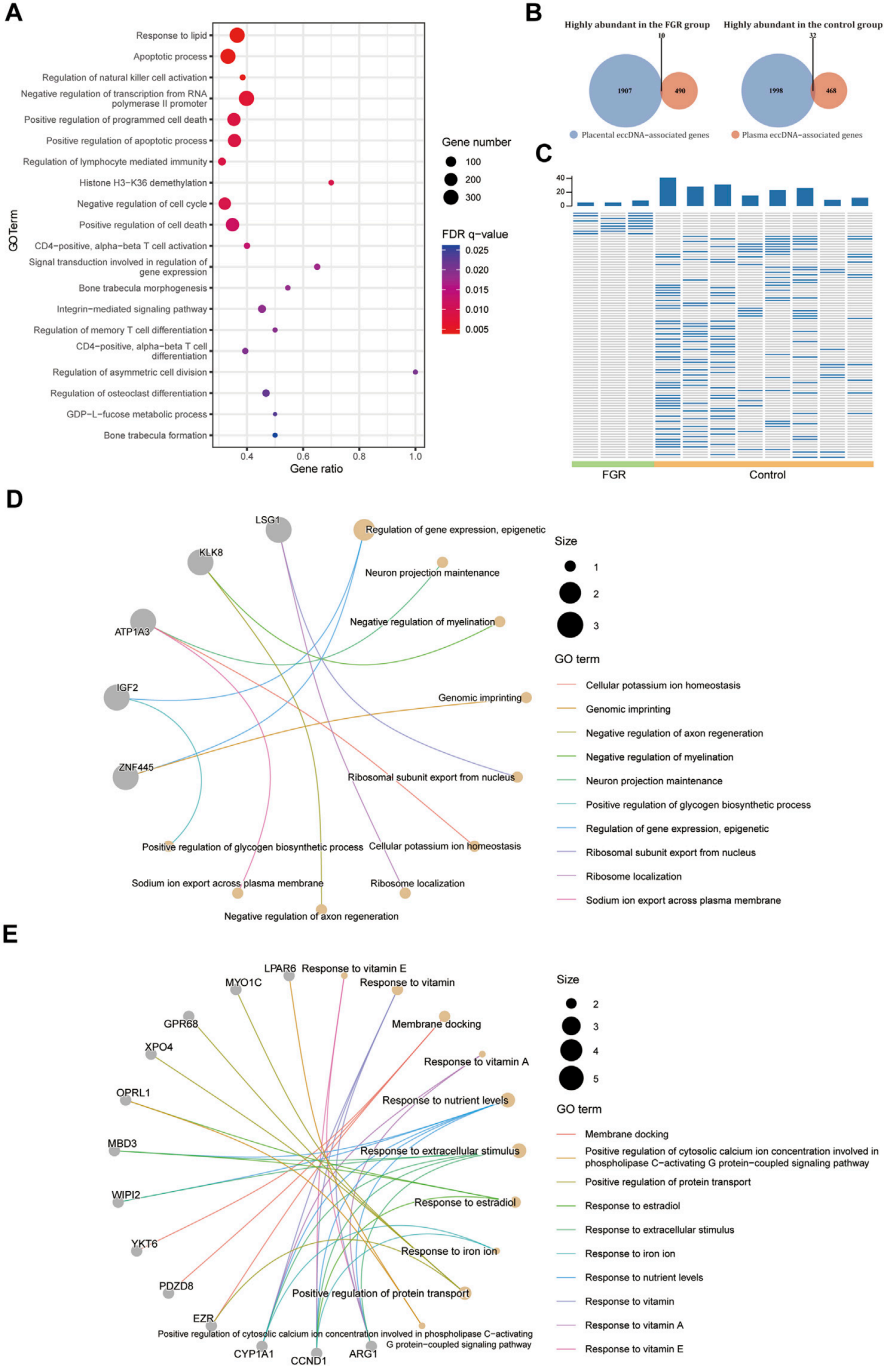
Differential abundance of plasma eccDNA–associated genes. **(A)** GO terms enriched in the differentially abundant eccDNA breakpoint flanking regions in plasma from the FGR and control groups. **(B)** Venn plot of highly abundant placental and plasma eccDNA–associated genes in the FGR and control groups. **(C)** Heat plot of common highly abundant eccDNA in each group. GO terms for highly abundant eccDNA–associated genes in plasma from the FGR **(D)** and control **(E)** groups.

As the changes in placenta eccDNA abundance in samples from FGR cases were associated closely with known biological processes and reproducible in new samples, we explored the suitability of plasma eccDNA molecules as effective biomarkers. We performed a differential analysis with plasma eccDNA data from three FGR cases and eight control cases. We identified 5,360 differentially abundant regions between the FGR and control groups, of which 5,354 were enriched in known motifs. These regions were submitted to GREAT (McLean et al., 2010) for cis-regulatory annotation and GO enrichment analysis. The differentially abundant regions were enriched in GO terms such as response to lipids, apoptotic processes, the regulation of natural killer cell activation, the negative regulation of transcription from the RNA polymerase II promoter, the positive regulation of programmed cell death, and the negative regulation of the cell cycle (Figure 5A), in accord with the biological process of FGR.

We screened for potential plasma eccDNA biomarkers of FGR. By intersecting the highly abundant plasma eccDNA– and differentially abundant placental eccDNA–associated genes in each group, we detected 10 highly abundant genes in the FGR group and 32 such genes in the control group (Figure 5B), which represented 10 and 95 highly abundant unique eccDNAs, respectively, in plasma from each group (Figure 5C; Supplementary Table S4). The highly abundant genes in the FGR group included *IGF2* and *ZNF445*, and were annotated to GO terms such as the epigenetic regulation of gene expression and genomic imprinting (Figure 5D); those in the control group included *MYO1C*, *GPR68*, *XPO4*, *EZR*, *CYP1A1*, *CCND1*, and *ARG1*, and were annotated to GO terms such as the response to vitamins, response to nutrient levels, and positive regulation of protein transport (Figure 5E). Collectively, these findings indicate that the plasma eccDNA spectrum in FGR is associated with placental eccDNA hallmarks of the disease and characteristic of associated biological changes.

## Discussion

In this study, we used eccDNA sequencing technologies to characterize the landscape of placental and maternal plasma eccDNA molecules. We showed that placental eccDNA was more enriched in functional elements than was plasma eccDNA. Multi-chromosomal-fragment eccDNA specifically existed in the placenta and was associated with enhancers. Differential placental and plasma eccDNA–associated genes were enriched in known pathways related to FGR in the FGR and control groups. The integrative analysis of placental and plasma eccDNA led to the identification of eccDNA candidates in maternal plasma as potential FGR biomarkers. Yang et al. (2021) performed research similar to ours on eccDNA in placentas affected by FGR, but in our study, we integrated sequencing of eccDNA from placenta and maternal plasma, discovered multi-chromosomal-fragment eccDNA in human placenta, and provide evidence for plasma eccDNA as a potential biomarker of pregnancy-associated complications.

We found that placenta and maternal plasma contain hundreds of thousands of eccDNA molecules. The size profiles of these molecules were similar to those reported previously (Sin et al., 2020), characteristic of size peaks expressed by folds of nucleosome plus linker DNA length. Multi-chromosomal-fragment eccDNA that we found in placenta has only been reported in cancer tissue (deCarvalho et al., 2018; Wu et al., 2019) and mouse embryonic stem cells (Wang et al., 2021). Using paired-end tag sequencing and droplet sequencing chromatin interaction assays, Zhu et al. (2021) demonstrated that widespread intra-eccDNA and genome-wide chromosomal interactions of eccDNA in cancer cells promoted tumor progression. We observed the enrichment of enhancer regions in multi-chromosomal-fragment eccDNA, correlated with genes in apoptosis, cell growth, and autophagy pathways. The enrichment of eccDNA in transcriptionally active regions and cis-regulatory elements suggests that eccDNA is involved in placenta functions.

Placental and plasma eccDNA tended to be enriched in genic elements, and this enrichment was more pronounced in placental eccDNA, implying a closer relationship with gene expression. In addition, placental eccDNA was highly abundant in SINE/Alu, SINE/MIR, srpRNA, and LTR/ERVL elements, which play numerous roles in the human genome and transcriptome in various phases of gene regulation (Wilhelm-Benartzi et al., 2012; Wang et al., 2015; Zhang et al., 2021b; Kellogg et al., 2022; Wang et al., 2022). Overall, functional linkage was common in placenta and plasma, but more distinct in placenta. The enrichment in functional elements indicates that eccDNA molecules are, on one hand, products of a series of biological processes, and on the other hand, may function in ways similar to their chromosomal counterparts.

Placental and plasma eccDNA breakpoints were enriched in specific motifs (ZNF460, ZNF384, Stat2, ZNF135, and SP5) relative to the whole genome background, probably reflecting common TFBSs as eccDNA generation hotspots and implying the presence of functioning cis-regulatory elements in eccDNA. Relative to that of plasma, the motif enrichment of the placental eccDNA breakpoints was associated with enrichment in the transcription factor families of more than three adjacent zinc fingers, AP-2, NF-κB–related factors, and Jun-related factors, suggesting a strong association with placental functions. For example, the NF-κB signaling pathway is critical for the maintenance of pregnancy (Sakowicz, 2018; Ariyakumar et al., 2021), and AP-2α and AP-2γ control syncytiotrophoblast-specific gene expression (Vaiman et al., 2013). Relative to that from controls, placental eccDNA from FGR cases was enriched in elements such as paired-related HD factors, NK homeobox, TALE-type HD factors, and FOX, suggesting dysregulation of the activities of these transcription factors.

We identified a group of placental eccDNA–associated genes that were differentially abundant in the FGR and control groups. In addition, the top enriched GO terms were immunologically and metabolically relevant, with results for the former similar to previous findings (Yang et al., 2021). These results are consistent with the occurrence of aberrant immune regulation (Sato, 2022) and fatty acid metabolism (Chen et al., 2022) in the pathogenesis of FGR and suggest that placental eccDNA plays a role in FGR. Six of the 10 top differentially abundant placental eccDNA molecules were successfully validated by Sanger sequencing, a non-optimal result but similar to those obtained in previous research (Kumar et al., 2020; Cen et al., 2022), which reflects the gap between next-generation sequencing technologies and Sanger sequencing as the traditional gold standard. We verified the significantly high abundance of eccDNA [*SBF1^circle 50,447,497–50,447,834^*] in new placenta samples with FISH, which targeted the specific junctional tags without the serial steps of isolation and enzyme treatment, and thus revealed the original abundance of the molecule. *SBF1* has been reported to act as a SET domain–dependent regulator involved in epigenetic regulatory mechanisms of growth and differentiation (Cui et al., 1998). It inhibits proliferation, induces apoptosis, and causes cell cycle arrest through the inhibition of the insulin-like growth factor 1–proliferating cell nuclear antigen pathway (Elgehama et al., 2021). Our results reflect the underlying functioning pathways in which placental eccDNA is involved. Paulsen et al. (2019) demonstrated that microDNA generates regulatory short RNAs independent of canonical promoters and may drive changes in gene expression and cell phenotypes. Thus, further investigation of the roles of eccDNA in physiological and pathological processes in different organs is needed.

CfDNA fragmentation patterns were demonstrated to reflect the actual structural interaction of transcription factors with DNA and to provide nucleosome footprints (Ulz et al., 2019). Similar to that observed in linear cfDNA, we found transcription factor binding motif enrichment around eccDNA breakpoints. Our functional annotation of the differential abundance of plasma eccDNA flanking regions in the FGR and control groups showed enrichment in GO terms related to the lipid response, apoptotic process, natural killer cell activation, and cell cycle. These results are consistent with pathological placental alterations occurring in FGR, such as the dysregulation of extrinsic apoptotic pathways involving natural killer cells and intrinsic apoptotic pathways in response to DNA damage (Kasture et al., 2021) and the impairment of lipid metabolism (Chao de la Barca et al., 2022). Although only approximately 10% of plasma eccDNA is derived from the placenta (Sin et al., 2020), the changes in plasma eccDNA that occur with FGR may be attributable in part to reactive maternal changes in physiology and metabolism. We further screened a few unique eccDNA molecules common in each group, whose annotated genes in the maternal circulation or placenta have been reported to modulate fetal growth. For example, *IGF2* has been reported to play a pivotal role in regulating fetal growth *via* maternal plasma (Sferruzzi-Perri et al., 2011) and placenta (Elsamadicy and Thompson, 2022), and placental *EZR* is involved in the transfer of nutrients from mother to fetus, thereby influencing fetal growth (Nishimura et al., 2014). Taken together, this evidence indicates that eccDNA is a potentially competitive type of molecule as a disease biomarker.

Our findings shed light on the characteristics of placental and maternal plasma eccDNA and the biological implications of the eccDNA spectrum in FGR. A major limitation of this study is the small sample; due to the heterogeneity of eccDNA (Zhu et al., 2017; Sin et al., 2022), we could not examine the exact same eccDNA loci in different samples in many cases. In addition, the blood samples were collected in the late gestational period and the biomarkers found may not be applicable for early FGR prediction. Although our study was preliminary and conducted with a small sample, it explored the potential of plasma eccDNA as a biomarker of pregnancy complications. The placenta is the source of pregnancy complications such as FGR and a major source of cfDNA in maternal plasma, and our comprehensive analysis of eccDNA in the placenta and plasma increased the power of our results. In the future, a larger cohort study is needed to establish optimal eccDNA biomarkers for the early prediction and diagnosis of pregnancy complications. Another limitation is that we did not perform a cell-line or animal experiment to confirm our findings, especially those regarding the potential functions of placental eccDNA. Additional research is required to explore the biological functions of multi-chromosomal-fragment and single-fragment eccDNA from placenta and other tissues. Furthermore, the use of single-molecule real-time sequencing to capture long reads and methylation patterns in eccDNA (Yu et al., 2021) and targeted gene knockout with CRISPR-CAS9 (Feng et al., 2022) would yield more detailed information.

## Conclusion

We performed an integrated characterization of eccDNA in placenta and maternal plasma from FGR cases. Extensive genomic annotation revealed a stronger correlation of placental than of plasma eccDNA with transcription activities. In addition, our findings suggest that placental multi-chromosomal-fragment eccDNA functions as an enhancer that alters gene expression. Furthermore, we showed that the placental eccDNA spectrum is associated closely with pathways related to the pathogenesis of FGR. Finally, our comprehensive analysis revealed potential eccDNA biomarkers of FGR; future studies of maternal plasma eccDNA signatures in the presence of pregnancy-associated diseases are warranted.

## Data Availability

The sequencing data for the participants who consented to data archiving have been deposited in the Sequence Read Archive under BioProject no. PRJNA935422. This study made use of data generated by The Chinese University of Hong Kong Circulating Nucleic Acids Research Group, as reported by Sin et al. in Proc Natl Acad Sci USA (doi.org/10.1073/pnas.1914949117).

## Abbreviations

FGR, fetal growth restriction; EFW, estimated fetal weight; cfDNA, cell-free DNA; eccDNA, extrachromosomal circular DNA; UTR, untranslated region; RCA, rolling circle amplification; UCSC, University of California at Santa Cruz; AME, analysis of motif enrichment; GO, gene ontology; FDR, false discovery rate; PCR, polymerase chain reaction; FISH, fluorescence *in situ* hybridization; FFPE, formalin-fixed paraffin-embedded; GREAT, Genomic Regions Enrichment of Annotations Tool; IGV, Integrative Genomics Viewer; SINE, short interspersed nuclear element; LTR, long terminal repeat; srpRNA, signal recognition particle RNA; MIR, mammalian-wide interspersed repeat; ERVL, endogenous retrovirus-like element; TFBS, transcription factor binding site; AP-2, activating enhancer-binding protein 2; NF-κB, nuclear factor kappa B; HD, homeodomain; TALE, three-amino-acid loop extension; FOX, forkhead box.
