# Supplementary material for: Integrative profiling of extrachromosomal circular DNA in placenta and maternal plasma provides insights into the biology of fetal growth restriction and reveals potential biomarkers
Source: Front Genet. 2023 Jul 5;14:1128082. doi: 10.3389/fgene.2023.1128082 (PMC10354665; doi:10.3389/fgene.2023.1128082)
Supplement: Supplementary file 1 [file DataSheet1.pdf]

Supplementary figures

Integrative profiling of extrachromosomal circular DNA in placenta and maternal plasma provides insights into the biology of fetal growth restriction and reveals potential biomarkers

Minhuan Lin[1], Yiqing Chen[1], Shuting Xia[1], Zhiming He[1], Xuegao Yu[2], Linhuan Huang[1], Shaobin Lin[1], Binrun Liang[3], Ziliang Huang[3], Shiqiang Mei[3], Dong Liu[2], Lingling Zheng[3]*, Yanmin Luo[1]*

[1] Department of Obstetrics & Gynecology, The First Affiliated Hospital of Sun Yat-sen University, Guangzhou, China

[2] Clinical Laboratory, The First Affiliated Hospital of Sun Yat-sen University, Guangzhou, China

[3] MOE Key Laboratory of Gene Function and Regulation, State Key Laboratory for Biocontrol, School of Life Sciences, Sun Yat-sen University, Guangzhou, China

Figure S1

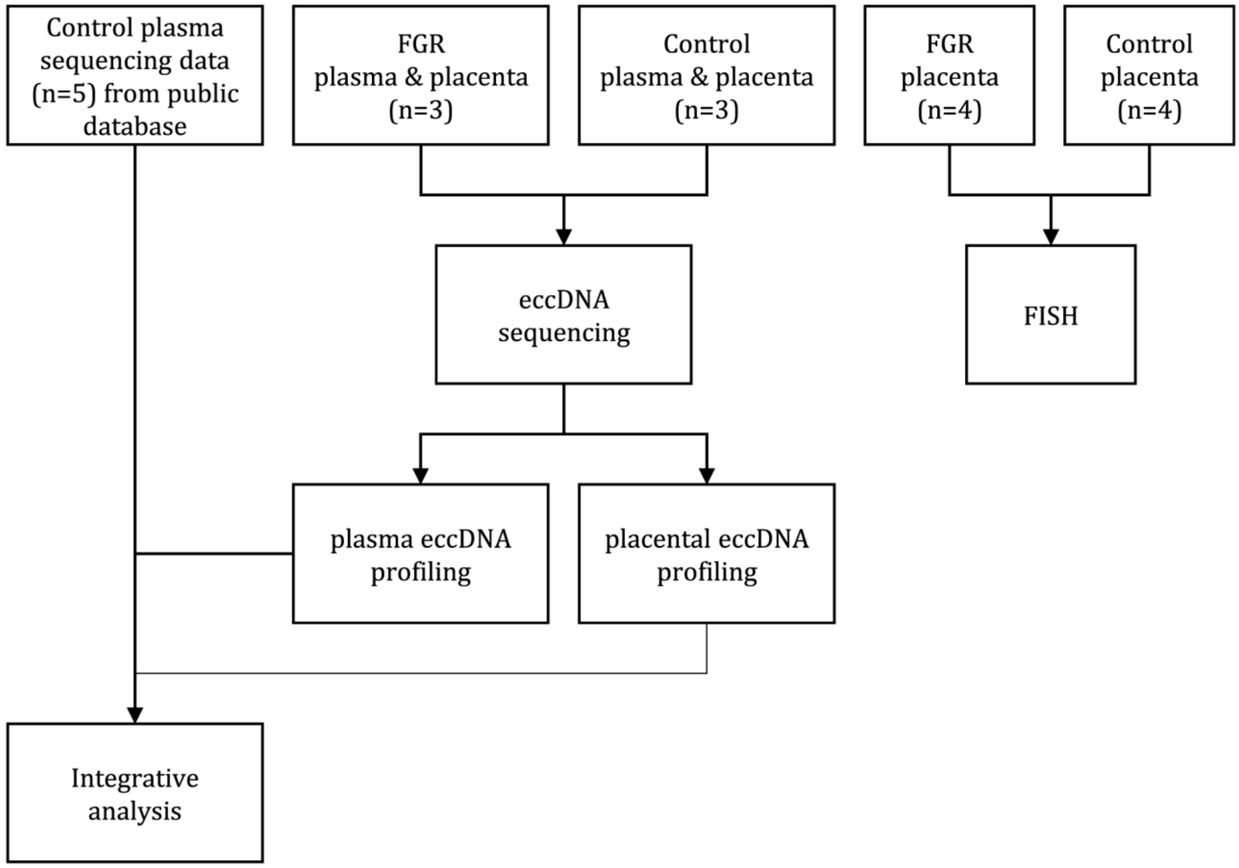

Flowchart of the study design.

Figure S2

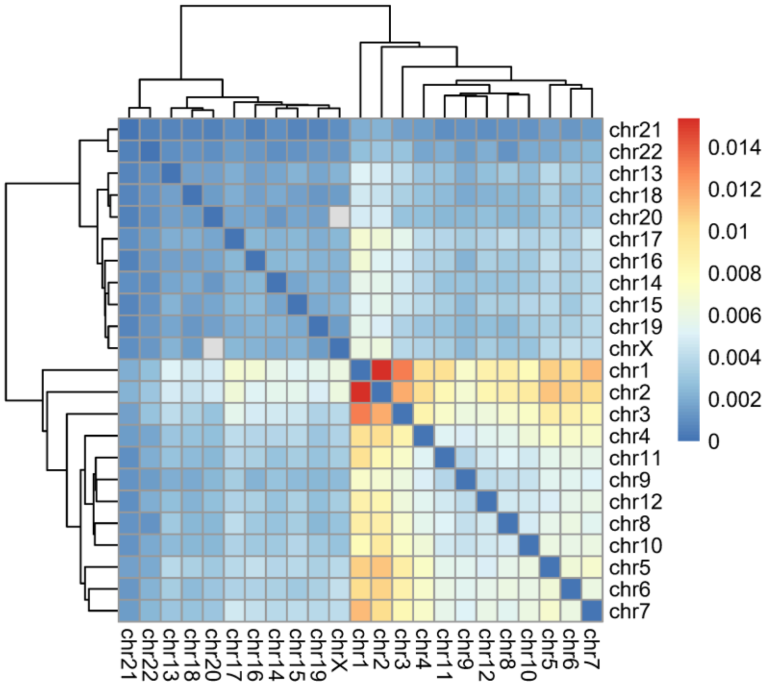

Heatmap of the intrachromosomal joining frequency of placental eccDNA, showing that fragments from chromosomes 1 and 2 were more likely to join multi-chromosomal eccDNA than were those from other chromosomes.

Figure S3

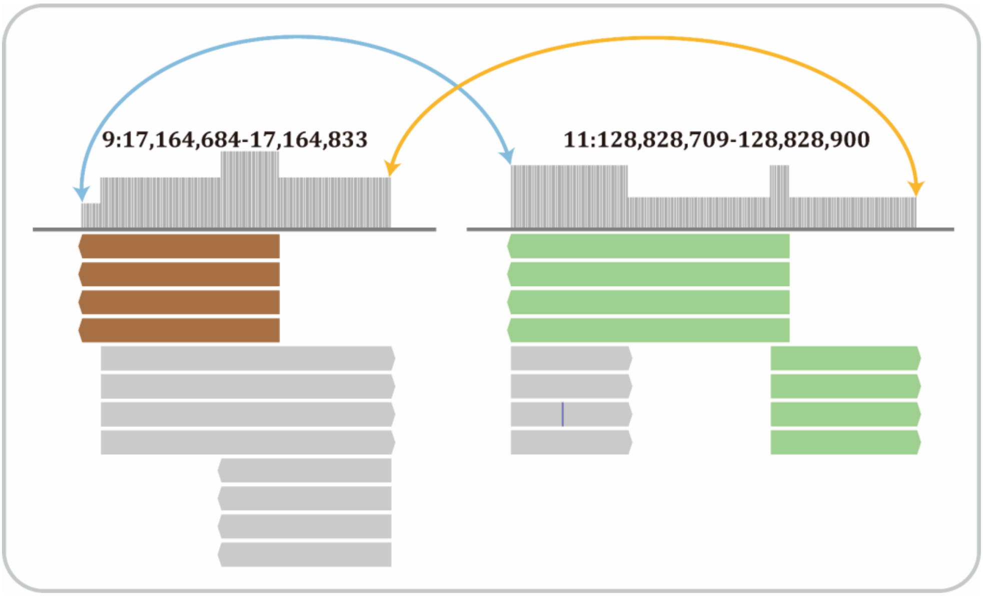

Example of two-fragment plasma eccDNA with supportive confident split reads visualized with the IGV.

Figure S4

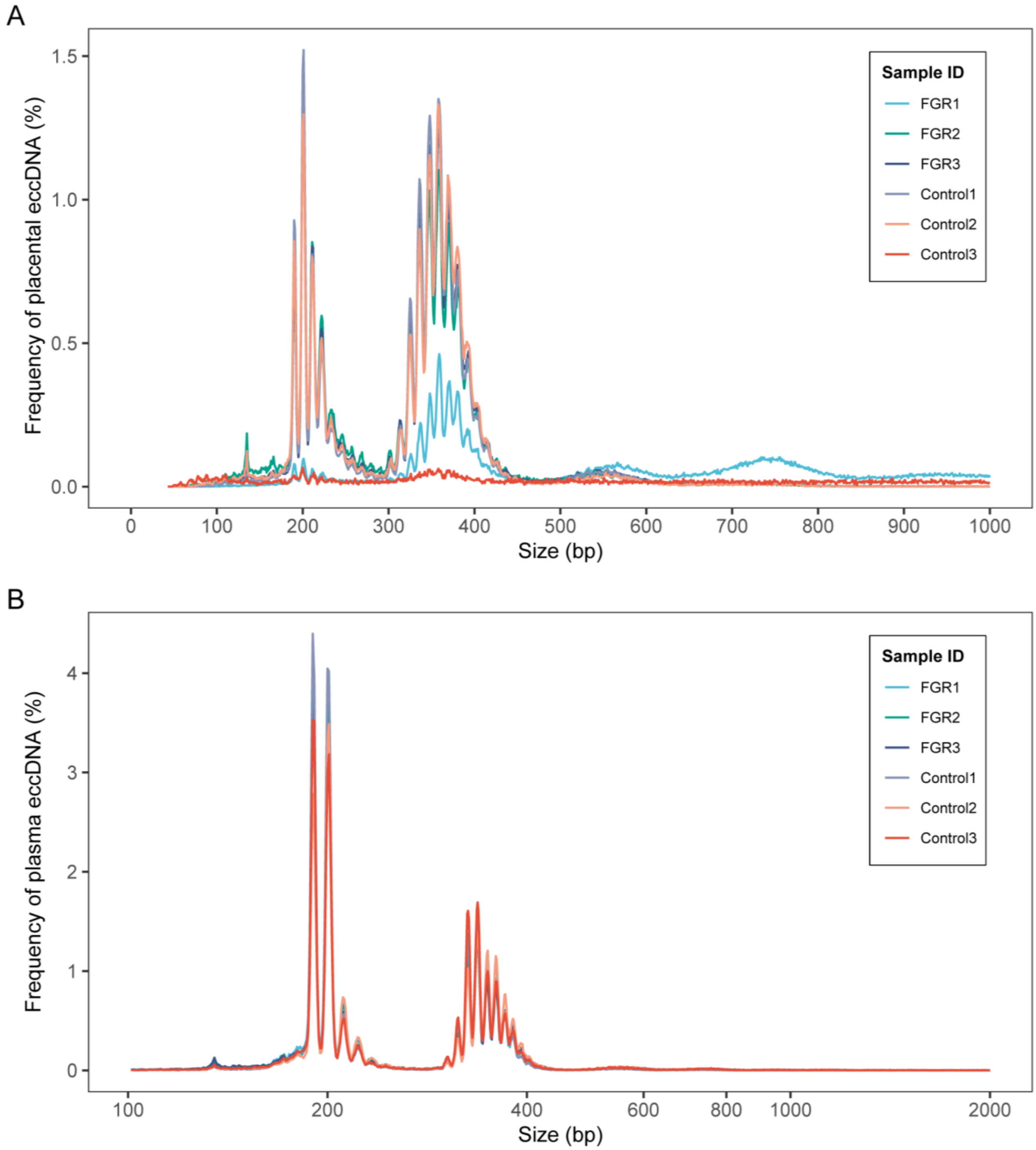

Size distributions of eccDNA identified in (A) placenta and (B) maternal plasma from three FGR and three control cases.

Figure S5

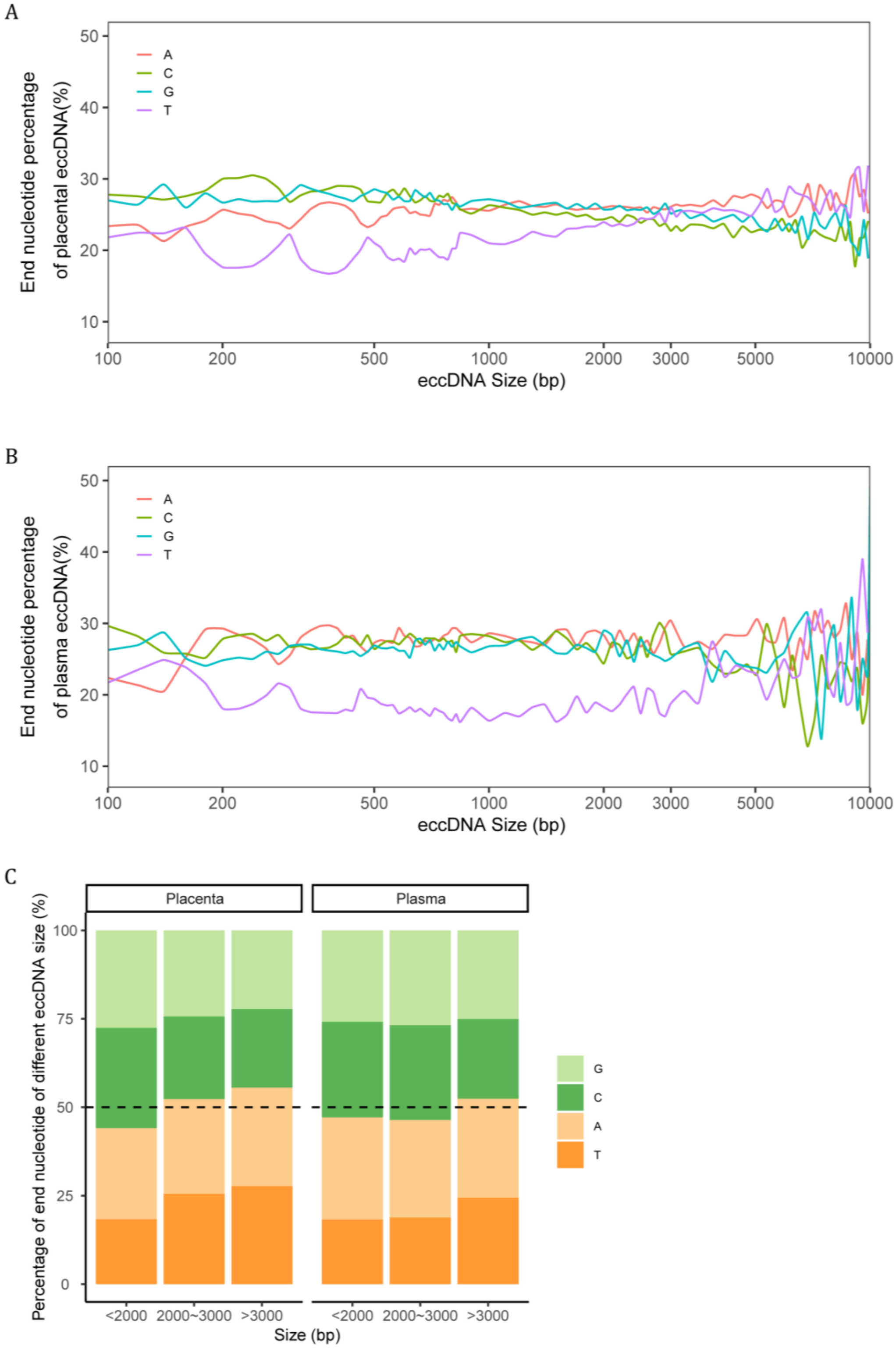

Percentages of eccDNA breakpoint nucleotides at the 5’ end of eccDNA genomic region across different eccDNA sizes in (A) placenta and (B) maternal plasma. (C) Stacked column chart with percentages of A/T/C/G end nucleotides of different eccDNA sizes in placenta and maternal plasma.

Figure S6

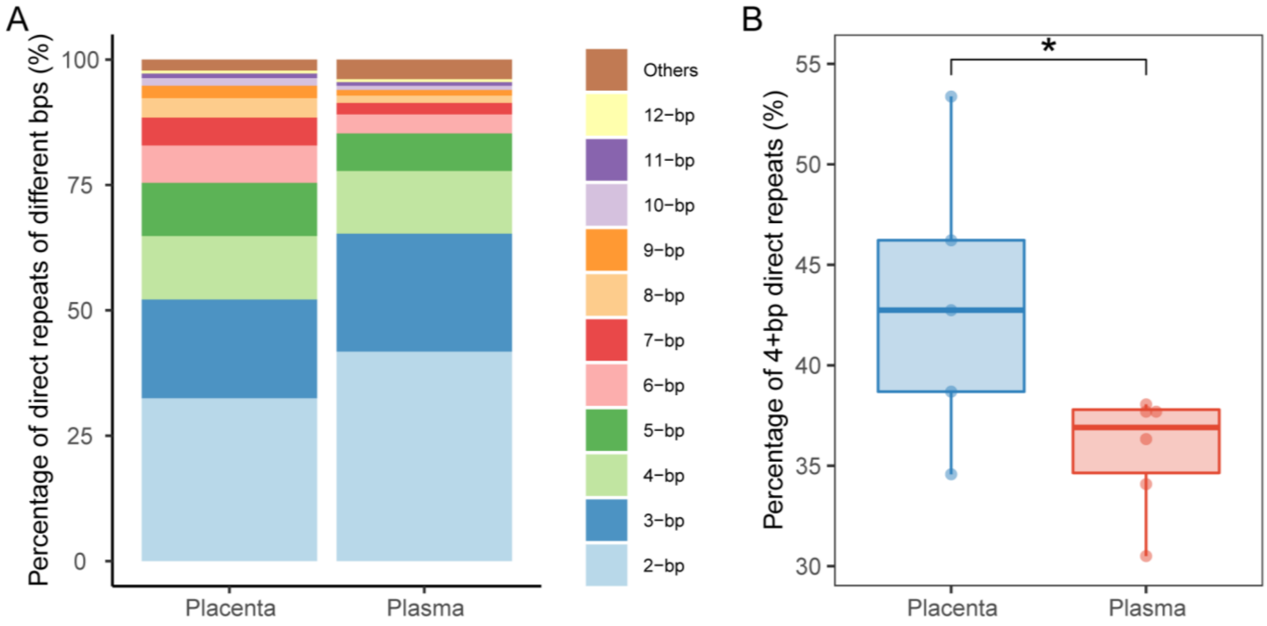

Percentages of flanking direct repeats of different (A) and 4~-bp (B) lengths in placenta and maternal plasma. *$p < 0.05$.

Figure S7

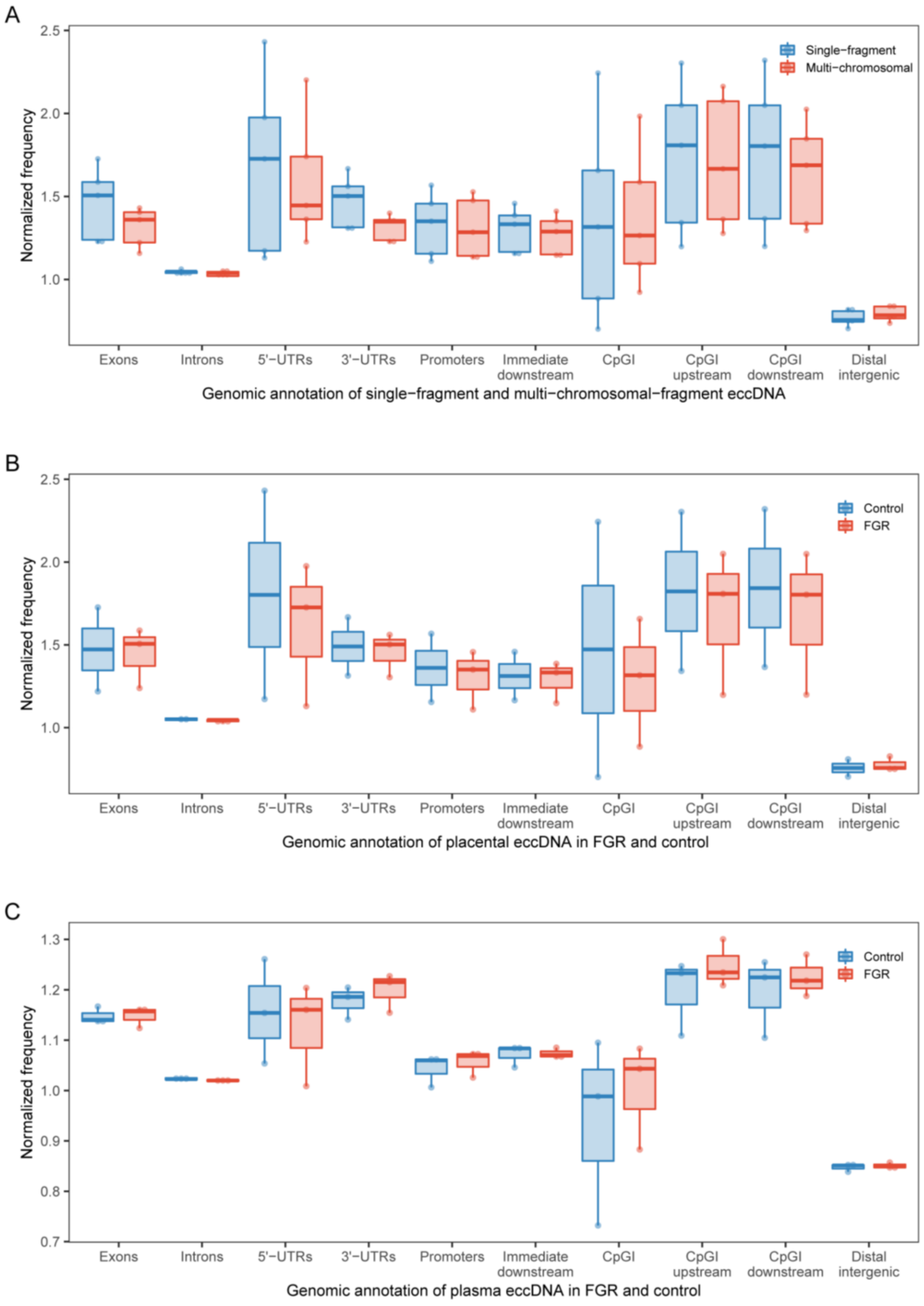

Normalized frequencies of structural annotation of breakpoints of (A) single-fragment and multi-chromosomal-fragment eccDNA in placenta, and of eccDNA in (B) placenta and (C) maternal plasma in the FGR and control groups.

Figure S8

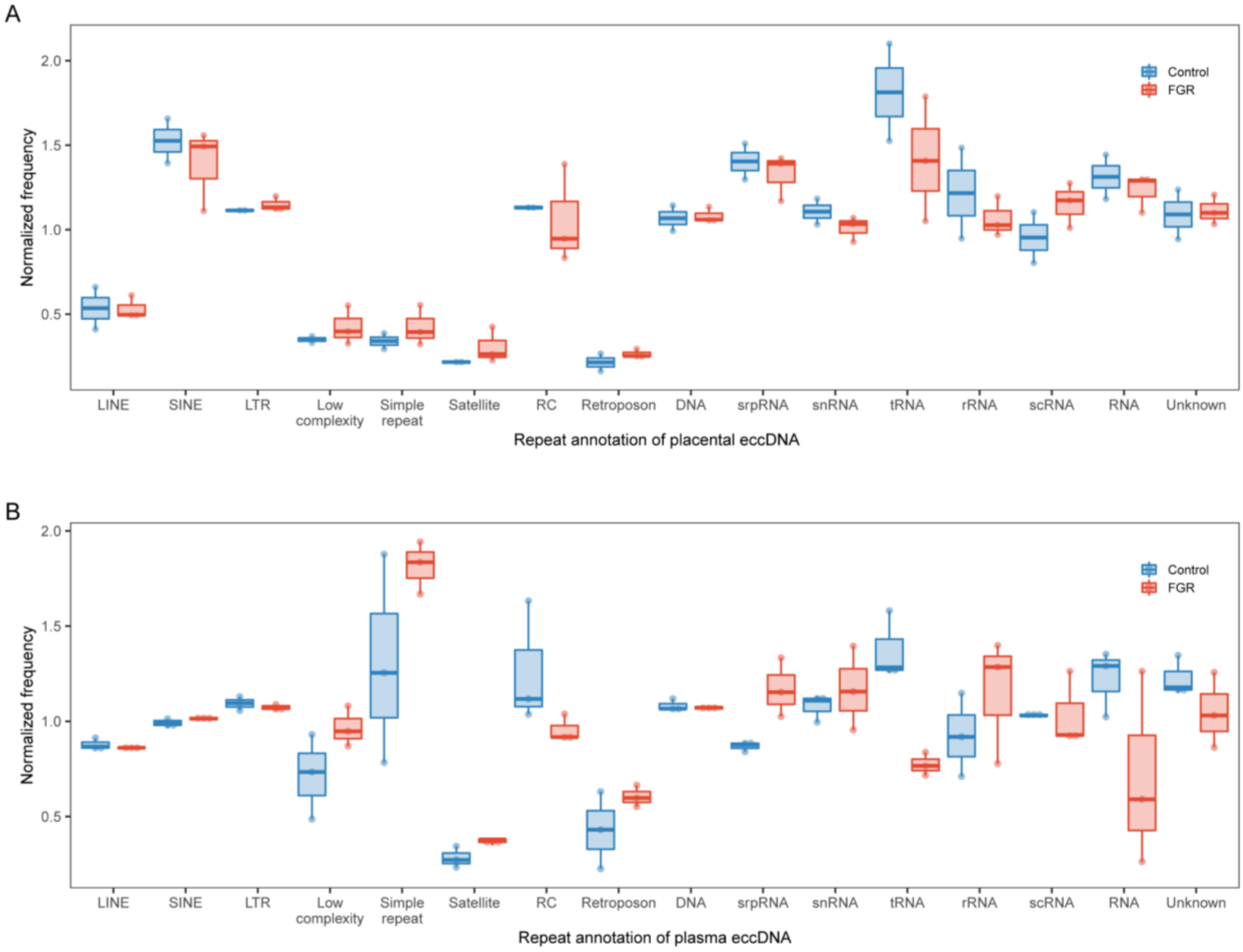

Normalized frequencies of repetitive annotation of breakpoints of (A) plasma and (B) placental eccDNA in the FGR and control groups.

Figure S9

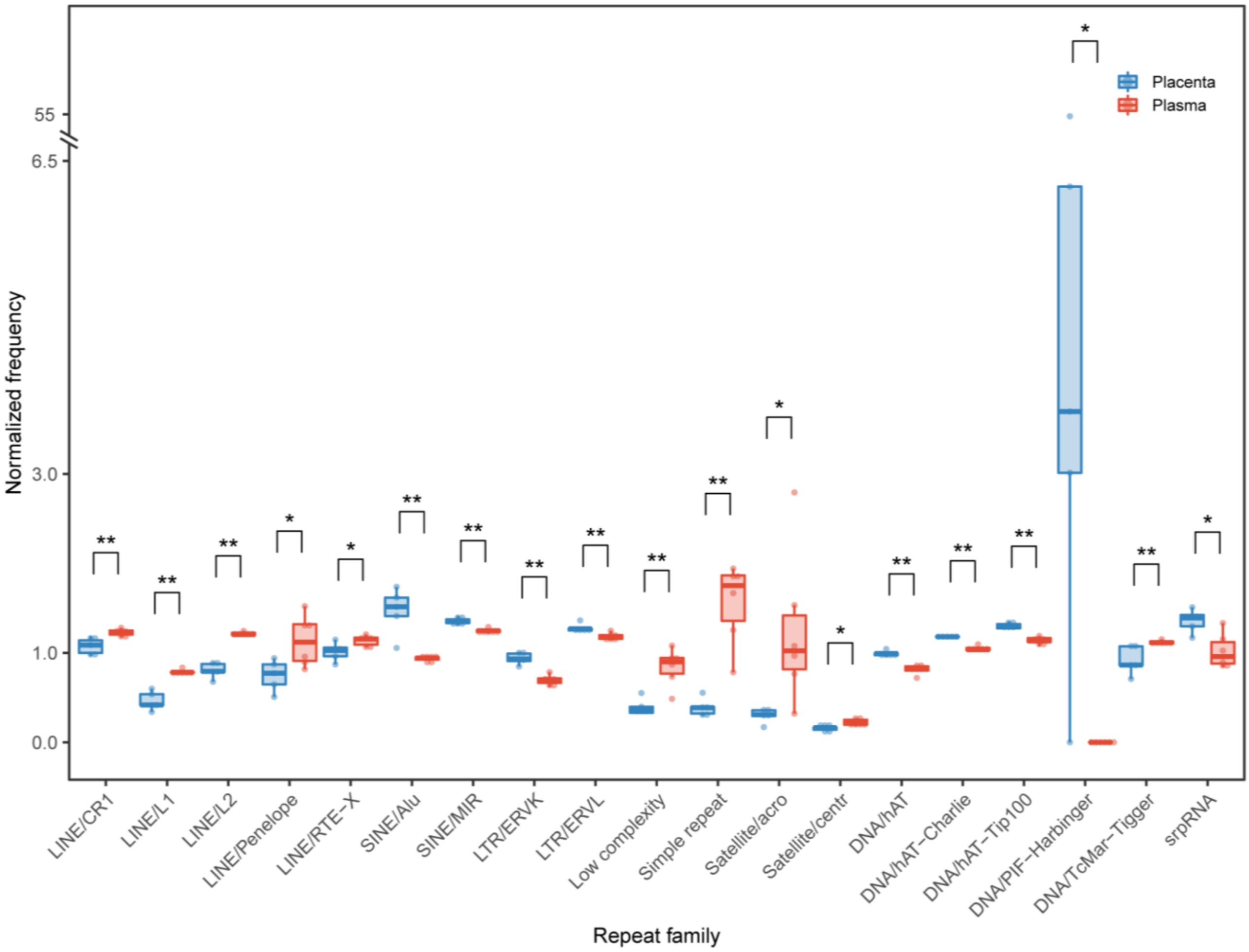

Normalized frequencies of significantly different repetitive families between placental and maternal plasma eccDNA. *$p < 0.05$, **$p < 0.01$.

Figure S10

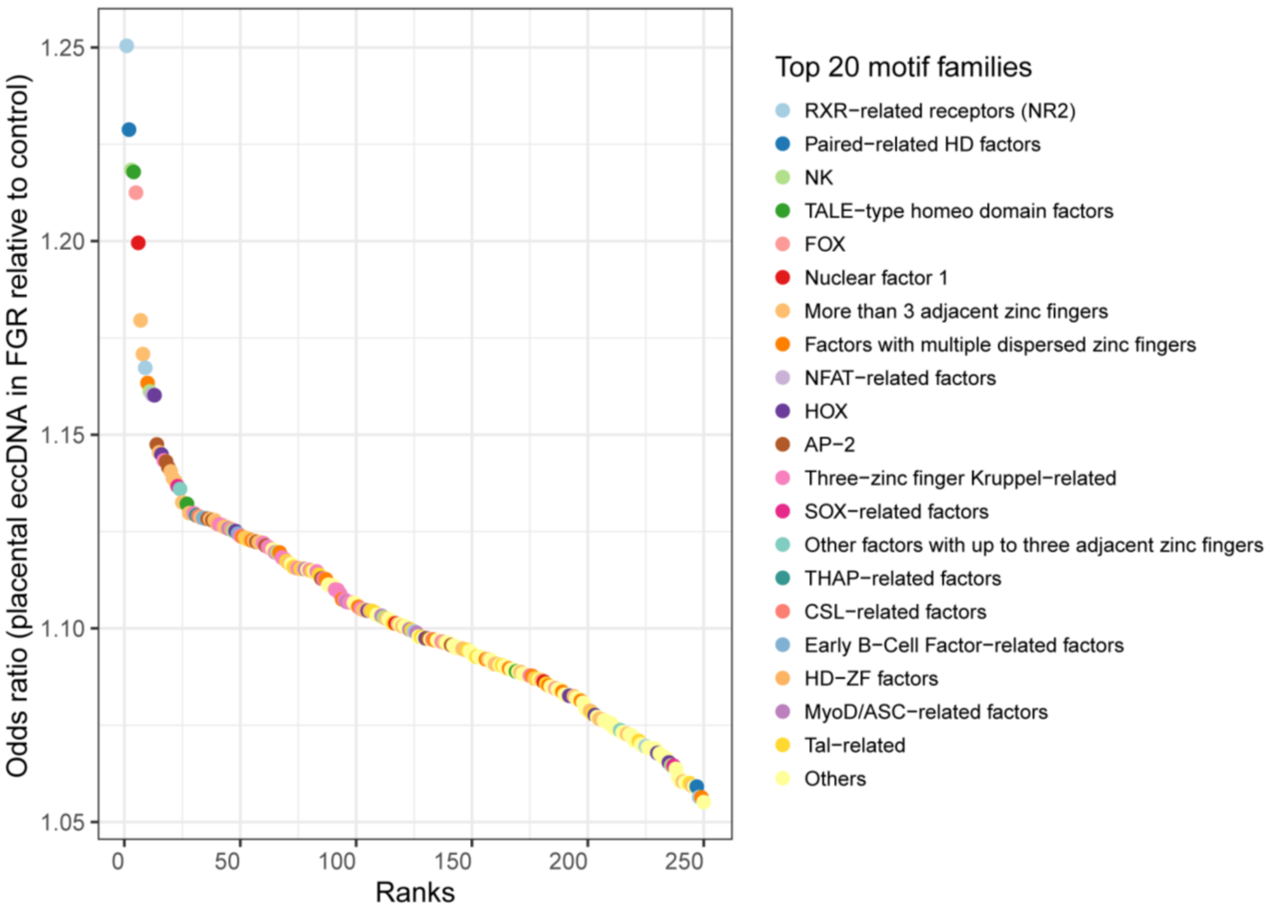

Top 20 significantly enriched transcription factor families in placental eccDNA from the FGR group relative to the control group.
